# Supplementary material for: scBridge embraces cell heterogeneity in single-cell RNA-seq and ATAC-seq data integration
Source: Nat Commun. 2023 Sep 28;14:6045. doi: 10.1038/s41467-023-41795-5 (PMC10539354; doi:10.1038/s41467-023-41795-5)
Supplement: Supplementary file 1 — Supplementary Information [file 41467_2023_41795_MOESM1_ESM.pdf]

Supplementary Information for scBridge embraces cell heterogeneity in single-cell RNA-seq and ATAC-seq data integration

Yunfan Li[1, †], Dan Zhang[2, †], Mouxing Yang[1], Dezhong Peng[1], Jun Yu[3], Yu Liu[4], Jiancheng Lv[1], Lu Chen[2], and Xi Peng[1, *]

[1]School of Computer Science, Sichuan University, Chengdu, Sichuan, China
[2]Key Laboratory of Birth Defects and Related Diseases of Women and Children of MOE, Department of Laboratory Medicine, State Key Laboratory of Biotherapy, West China Second University Hospital, Sichuan University, Chengdu, China
[3]School of Computer Science, Hangzhou Dianzi University, Hangzhou, Zhejiang, China
[4]School of Electronic and Information Engineering, Naval Aviation University, Yantai, Shandong, China
[*]pengx.gm@gmail.com
[†]equal contribution

Supplementary Figures

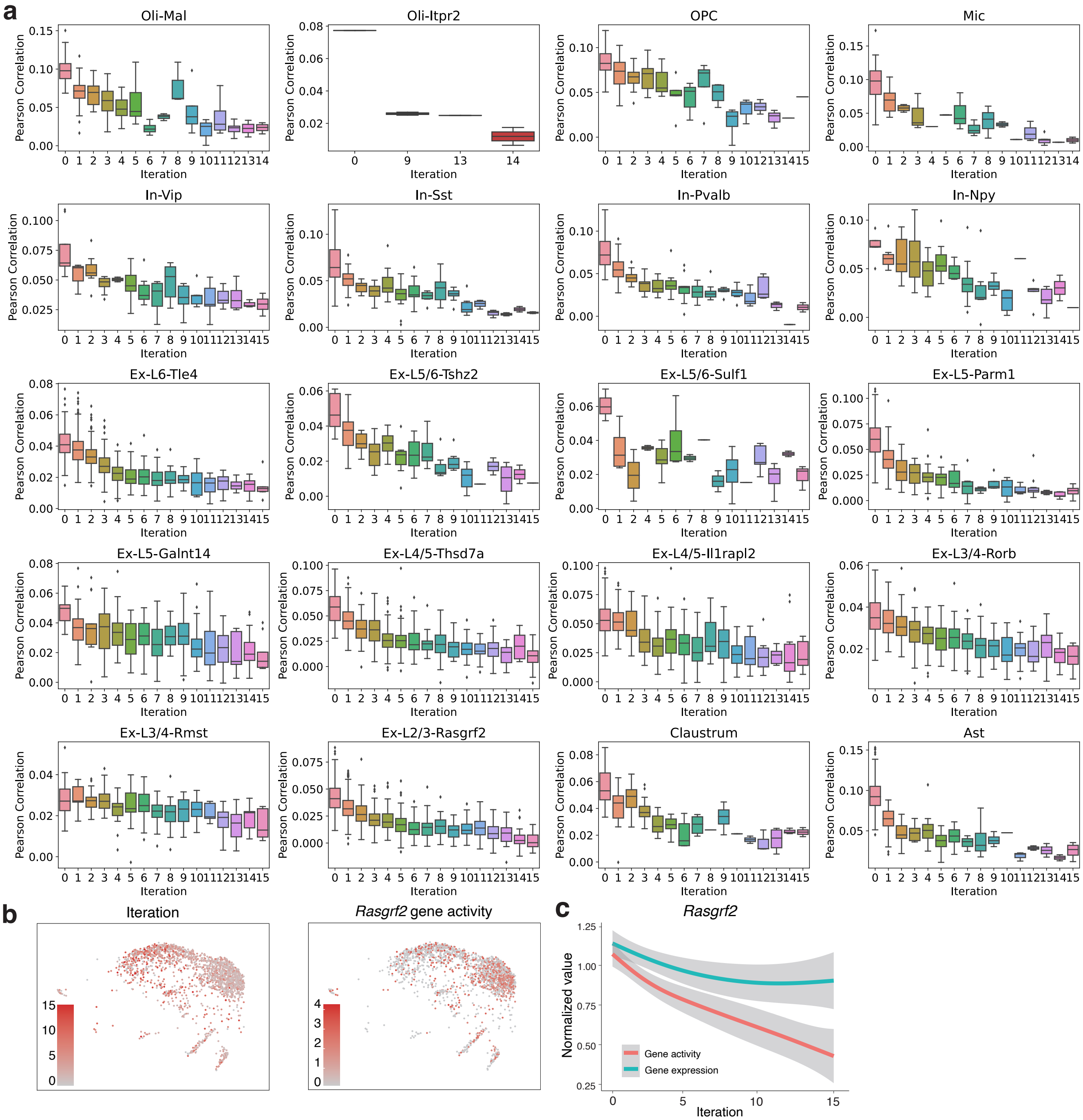

Supplementary Figure 1. **a**, The Pearson correlation score between scRNA-seq and the selected scATAC-seq cells of 20 classes in different iterations on the SNARE-seq dataset. Specifically, we first averaged the normalized gene expression of scRNA-seq cells for each type. Then, we computed the Pearson correlation score between the normalized gene activity of each scATAC-seq cell and the mean gene expression according to the predicted cell type. Each boxplot ranges from the upper and lower quartiles with the median as the horizontal line and whiskers extend to 1.5 times the interquartile range. **b**, Iteration and the gene activity of marker gene *Rasgrf2*[1] projected on the UMAP plot of Ex-L2/3-Rasgrf2 cells of scATAC-seq. **c**, Dynamic change of *Rasgrf2* gene expression and gene activity in Ex-L2/3-Rasgrf2 cells of scRNA-seq and scATAC-seq, respectively. The gray area denotes the standard error.

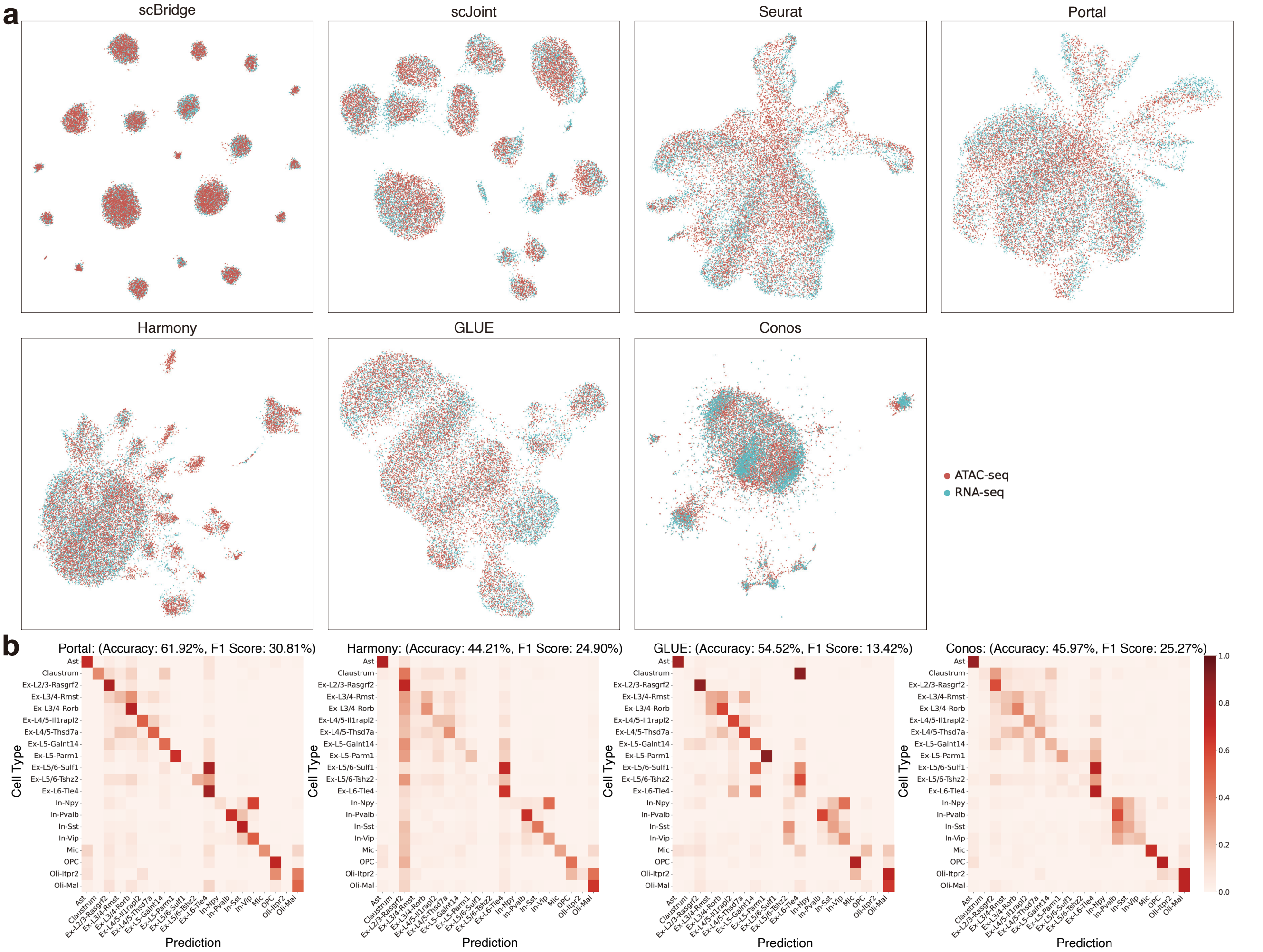

Supplementary Figure 2. **a**, UMAP visualization of the joint embedding obtained by scBridge and six baseline methods on the SNARE-seq dataset. Cells are colored by omics. **b**, Label transfer agreements between the original label and the label transferred by Portal, Harmony, GLUE, and Conos. A clearer diagonal structure indicates better label transfer performance.

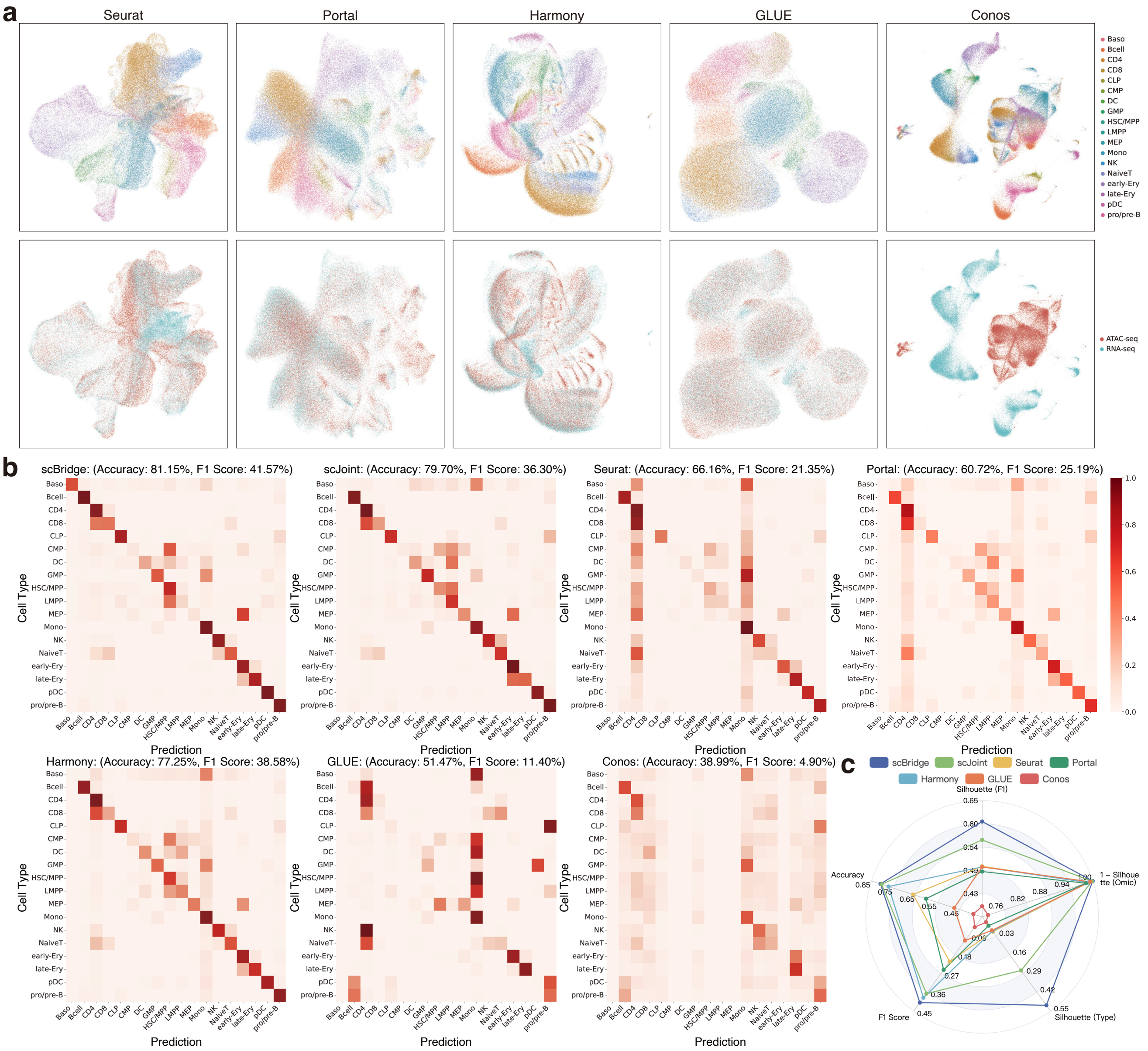

Supplementary Figure 3. **a**, UMAP visualization of the joint embedding obtained by Seurat, Portal, Harmony, GLUE, and Conos on the SHARE-seq dataset. The first and second rows show cells colored by types and omics, respectively. **b**, Label transfer agreements between the original label and the label transferred by scBridge and six baselines. A clearer diagonal structure indicates better label transfer performance. **c**, Quantitative evaluation in terms of the joint embedding quality and label transfer accuracy on the SHARE-seq dataset. Source data are provided as a Source Data file.

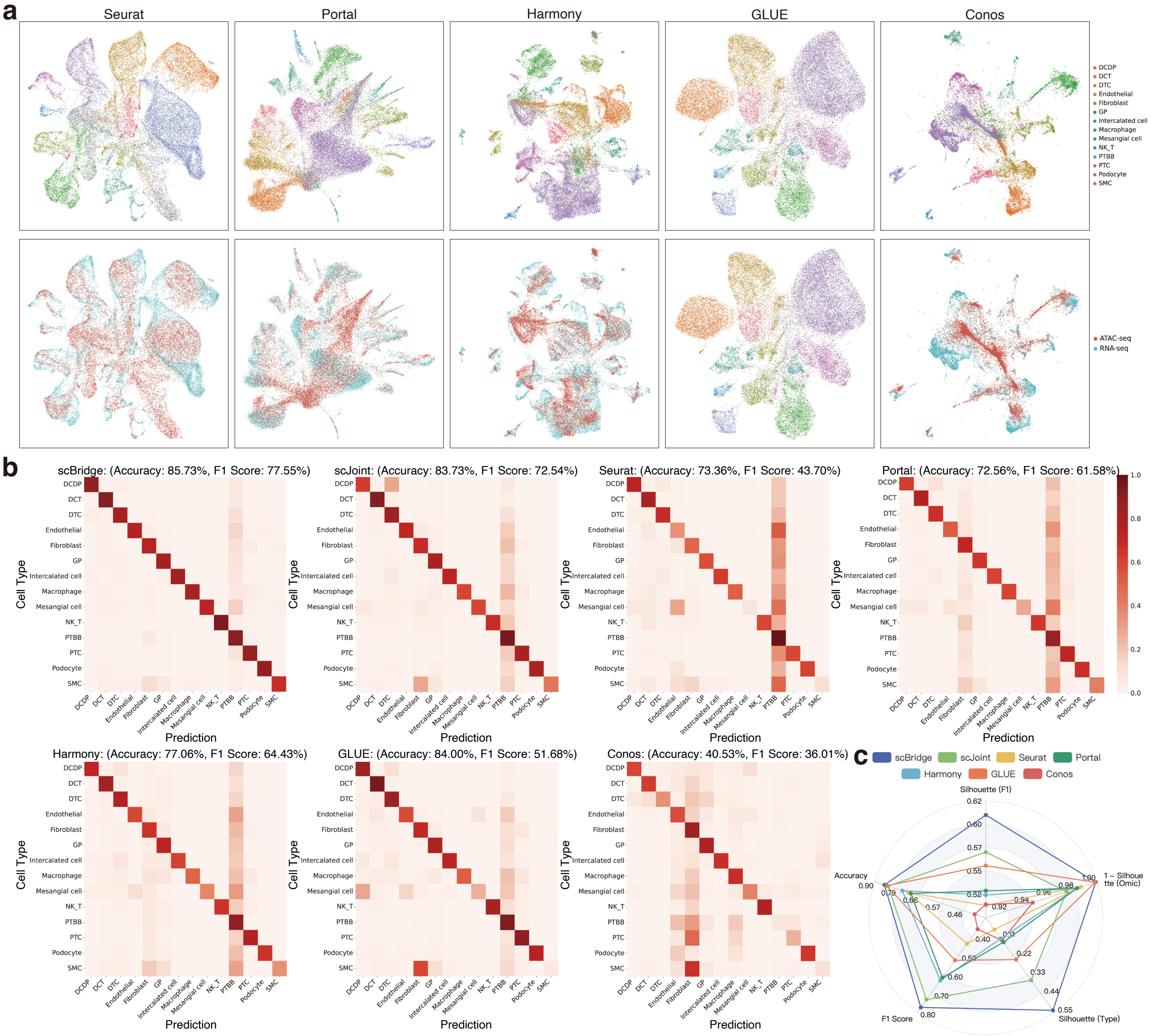

Supplementary Figure 4. **a**, UMAP visualization of the joint embedding obtained by Seurat, Portal, Harmony, GLUE, and Conos on the 10x Multiome dataset. The first and second rows show cells colored by types (DCDP: Distal collecting duct principal cell, DCT: Distal convoluted tubule cell, DTC: Distal tubule cell, PTC: Proximal tubule cell, SMC: Smooth muscle cell, GP: Glomerular podocyte, PTBB: Proximal tubule brush border cell) and omics, respectively. **b**, Label transfer agreements between the original label and the label transferred by scBridge and six baselines. A clearer diagonal structure indicates better label transfer performance. **c**, Quantitative evaluation in terms of the joint embedding quality and label transfer accuracy on the 10x Multiome dataset. Source data are provided as a Source Data file.

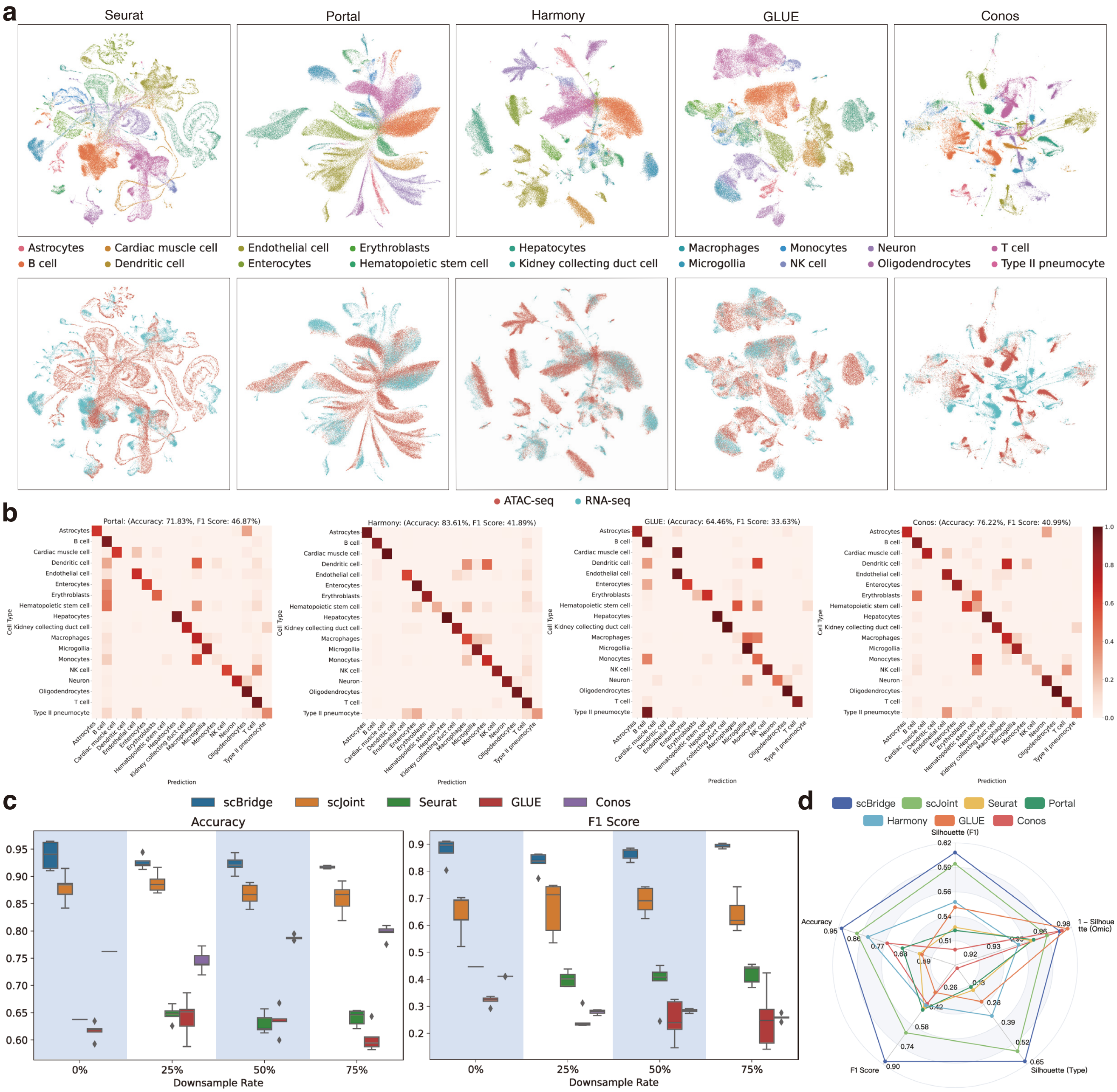

Supplementary Figure 5. **a**, UMAP visualization of Seurat, Portal, Harmony, GLUE, and Conos on the mouse atlas dataset. The first and second rows show cells colored by types and omics, respectively. **b**, Label transfer agreements between the original label and the label transferred by Portal, Harmony, GLUE, and Conos. A clearer diagonal structure indicates better label transfer performance. **c**, The label transfer accuracy and F1-score of the tested methods on mouse atlas, where 100%, 75%, 50%, and 25% annotated scRNA-seq data are used. Each boxplot ranges from the upper and lower quartiles with the median as the horizontal line and whiskers extend to 1.5 times the interquartile range. Source data are provided as a Source Data file. **d**, Quantitative evaluation in terms of the joint embedding quality and label transfer accuracy on the Mouse Atlas dataset. Source data are provided as a Source Data file.

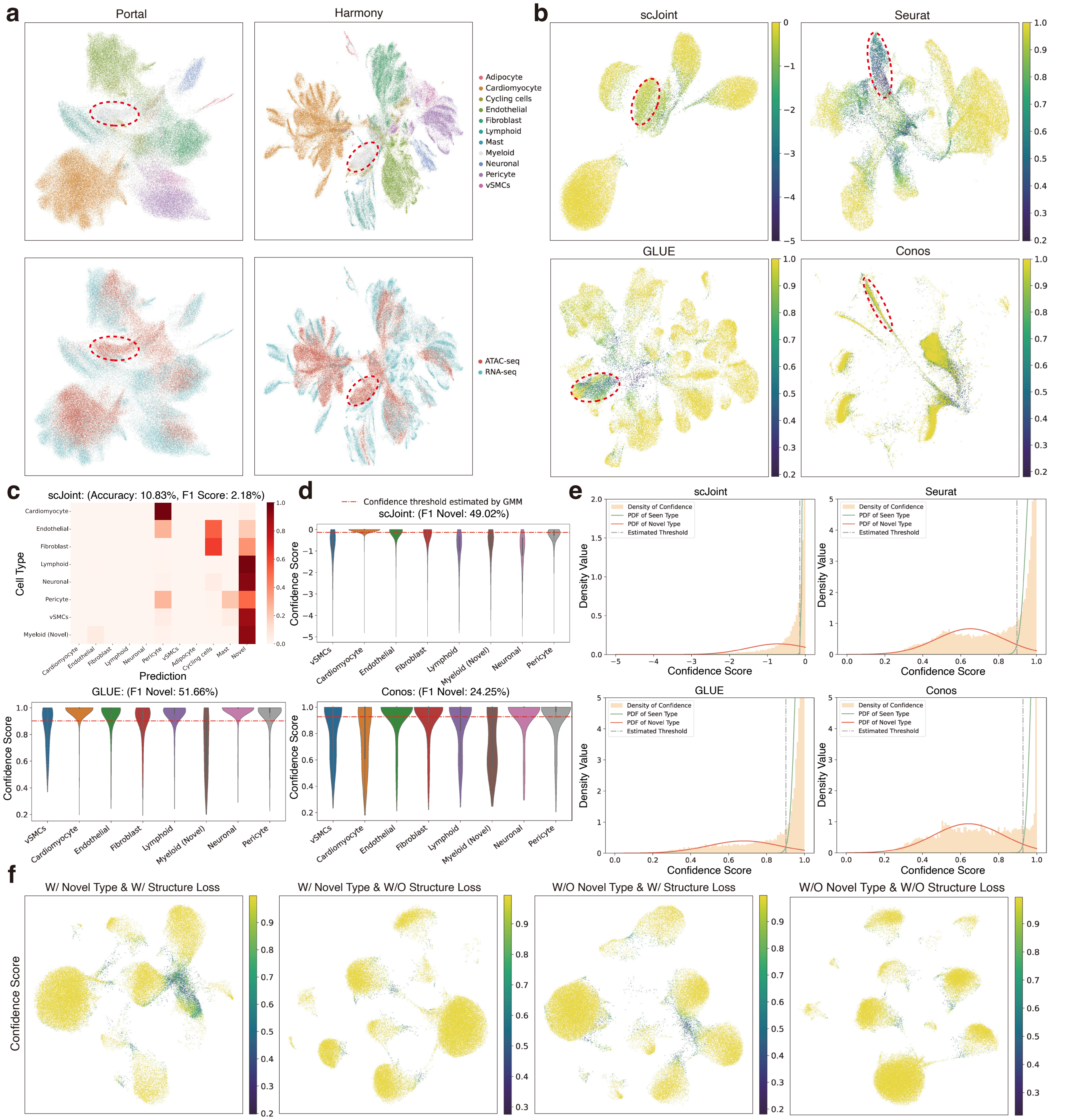

Supplementary Figure 6. **a**, UMAP visualization of Portal and Harmony on the human myocardial infarction data with novel types. The first and second rows show cells colored by types and omics, respectively. **b**, UMAP visualization of scATAC-seq cells obtained by scJoint, Seurat, GLUE, and Conos, where cells are colored by the confidence score. **c**, The label transfer results of scJoint. Cells with a confidence score below the GMM-estimated threshold are considered novel. **d**, The confidence score for different types of scATAC-seq cells predicted by scJoint, GLUE, and Conos. **e**, The novel type threshold of scJoint, Seurat, GLUE, and Conos, which is estimated by applying a two-component GMM on the confidence score. **f**, UMAP visualization of scATAC-seq cells obtained by scBridge, where cells colored by the confidence score on four cases. Namely, from left to right, enabling structure loss on data with novel cell type, disabling structure loss on data with novel cell type, enabling structure loss on data without novel cell type, and disabling structure loss on data without novel cell type.

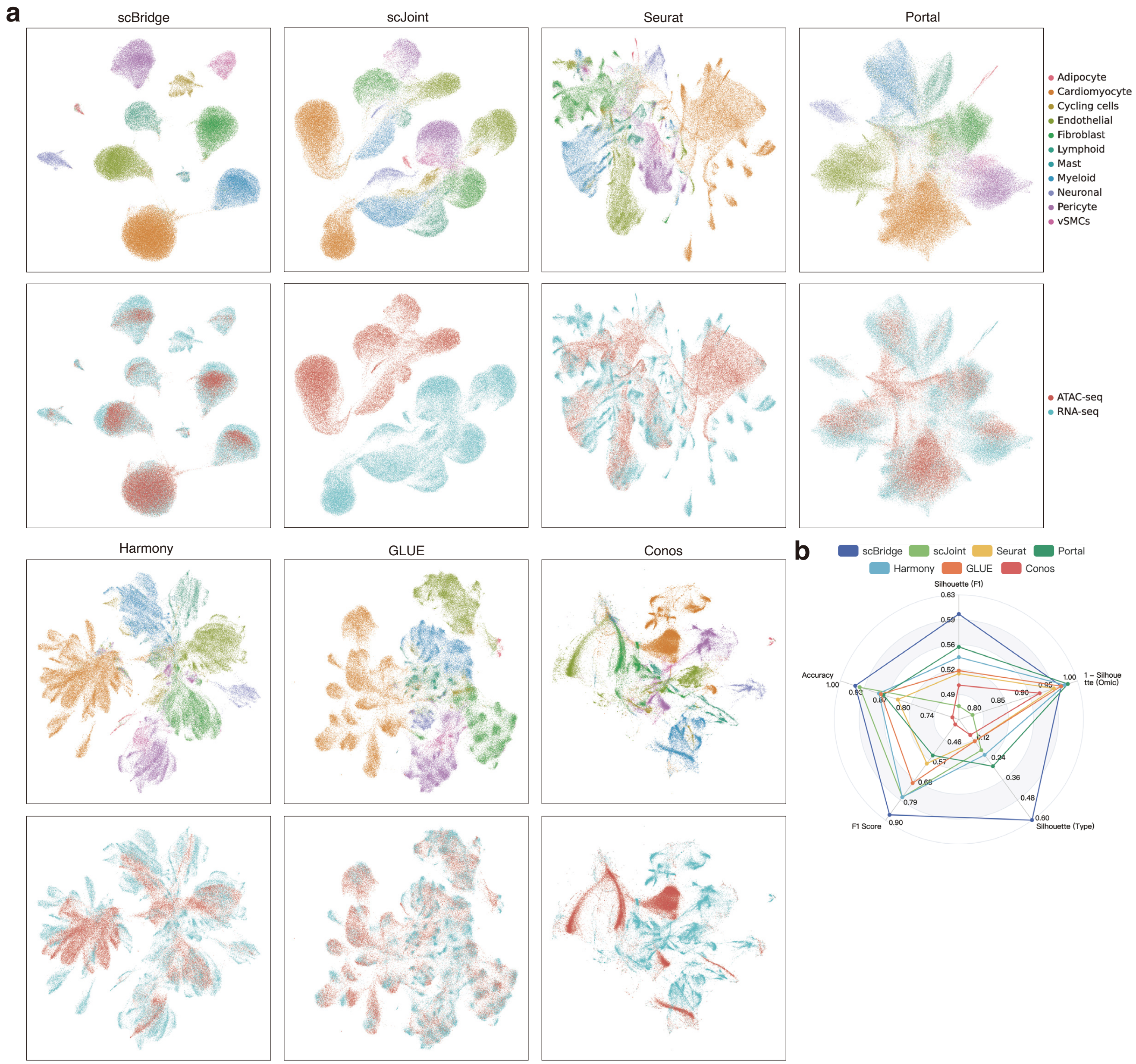

Supplementary Figure 7. **a**, UMAP visualization of scBridge and four baseline methods on the human myocardial infarction data. The first and second rows show cells colored by types and omics, respectively. **b**, Radar plot of five quantitative metrics to evaluate the integration quality and label transfer accuracy for scBridge and six baseline methods. Source data are provided as a Source Data file.

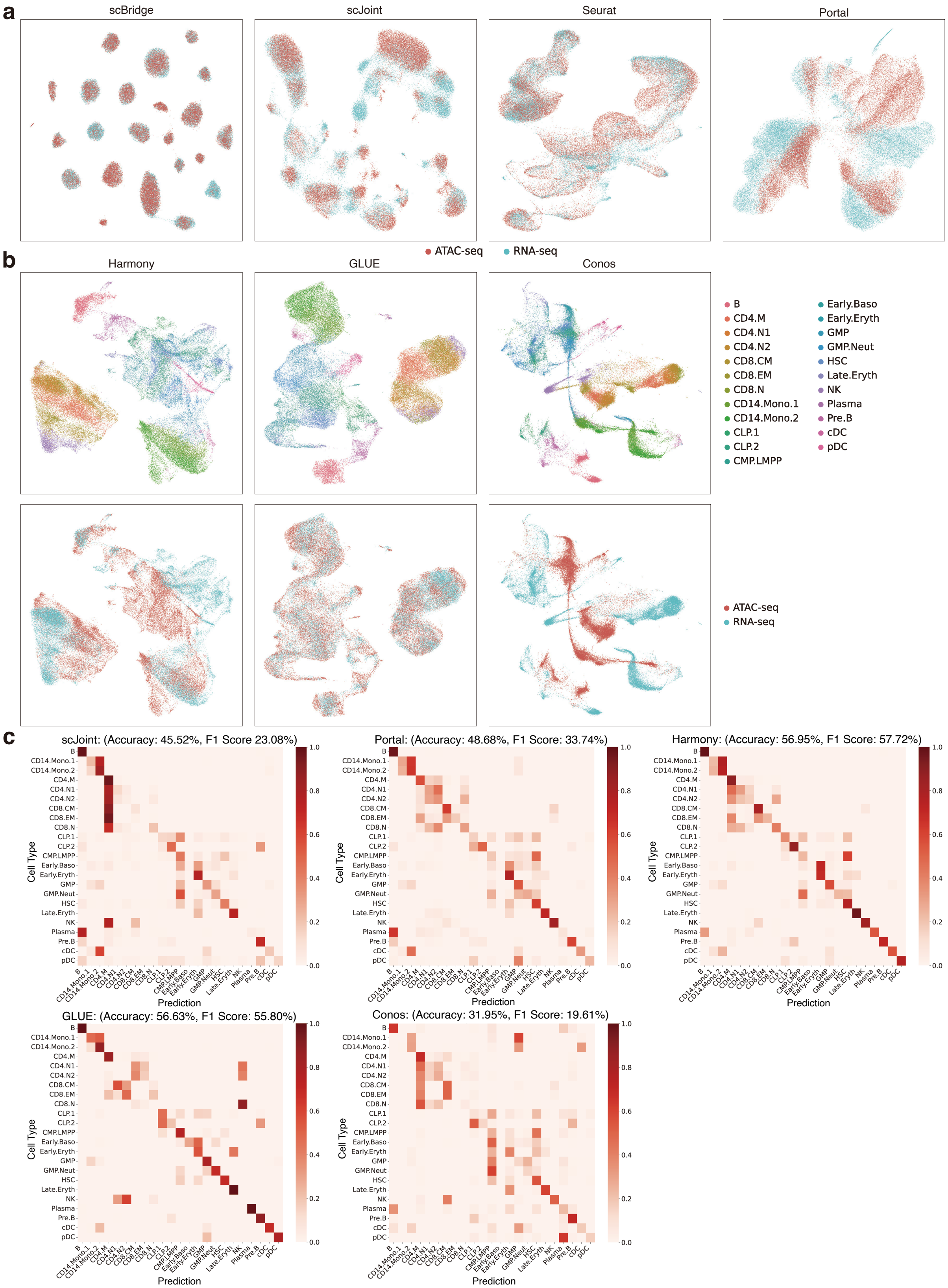

Supplementary Figure 8. **a**, UMAP visualization of scBridge, scJoint, Seurat, and Portal on the human hematopoiesis dataset under 75% dropout on scRNA-seq data. Cells are colored by omics. **b**, UMAP visualization of Harmony, GLUE, and Conos. The first and second rows show cells colored by types and omics, respectively. **c**, Label transfer agreements between the original label and the label transferred by scJoint, Portal, Harmony, GLUE, and Conos.

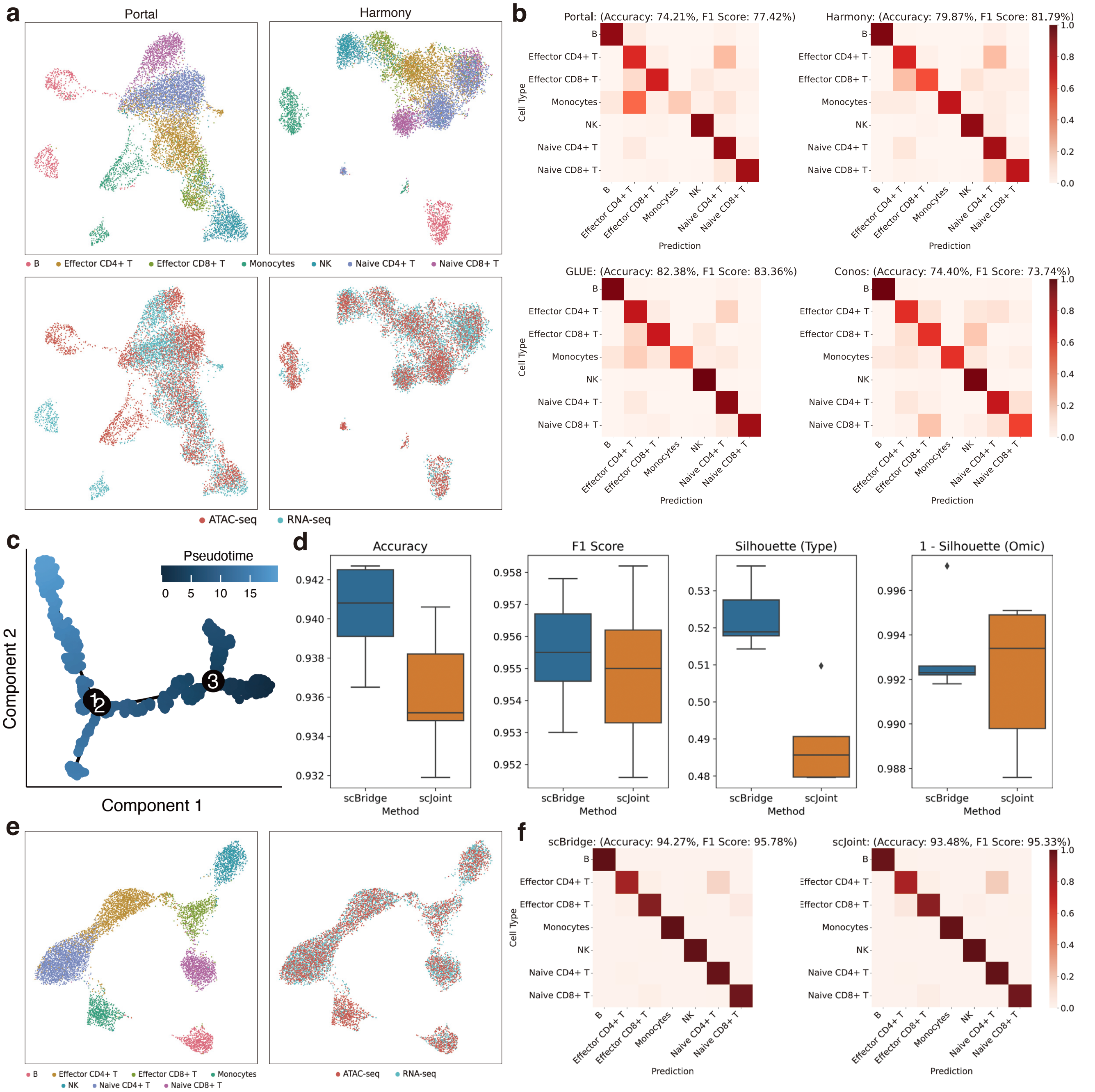

Supplementary Figure 9. **a**, UMAP visualization of the joint embedding obtained by Portal and Harmony on human PBMC data. The first and second rows show cells colored by types and omics, respectively. **b**, Label transfer agreements between the original label and the label transferred by Portal, Harmony, GLUE, and Conos. A clearer diagonal structure indicates better performance.**c**, Pseudotime projected on the monocle reduced space. **d**, Boxplot of the quantitative performances of scBridge and scJoint on integrating the protein data of human PBMC, in terms of label transfer accuracy, F1-score, and embedding silhouette score based on cell types and omics. Each boxplot ranges from the upper and lower quartiles with the median as the horizontal line and whiskers extend to 1.5 times the interquartile range. Source data are provided as a Source Data file. **e**, UMAP visualization of scBridge's integration result on the protein data, colored by cell types and omics respectively. **f**, Heatmap of the agreement between the original label and the label predicted by scBridge and scJoint on the protein data.

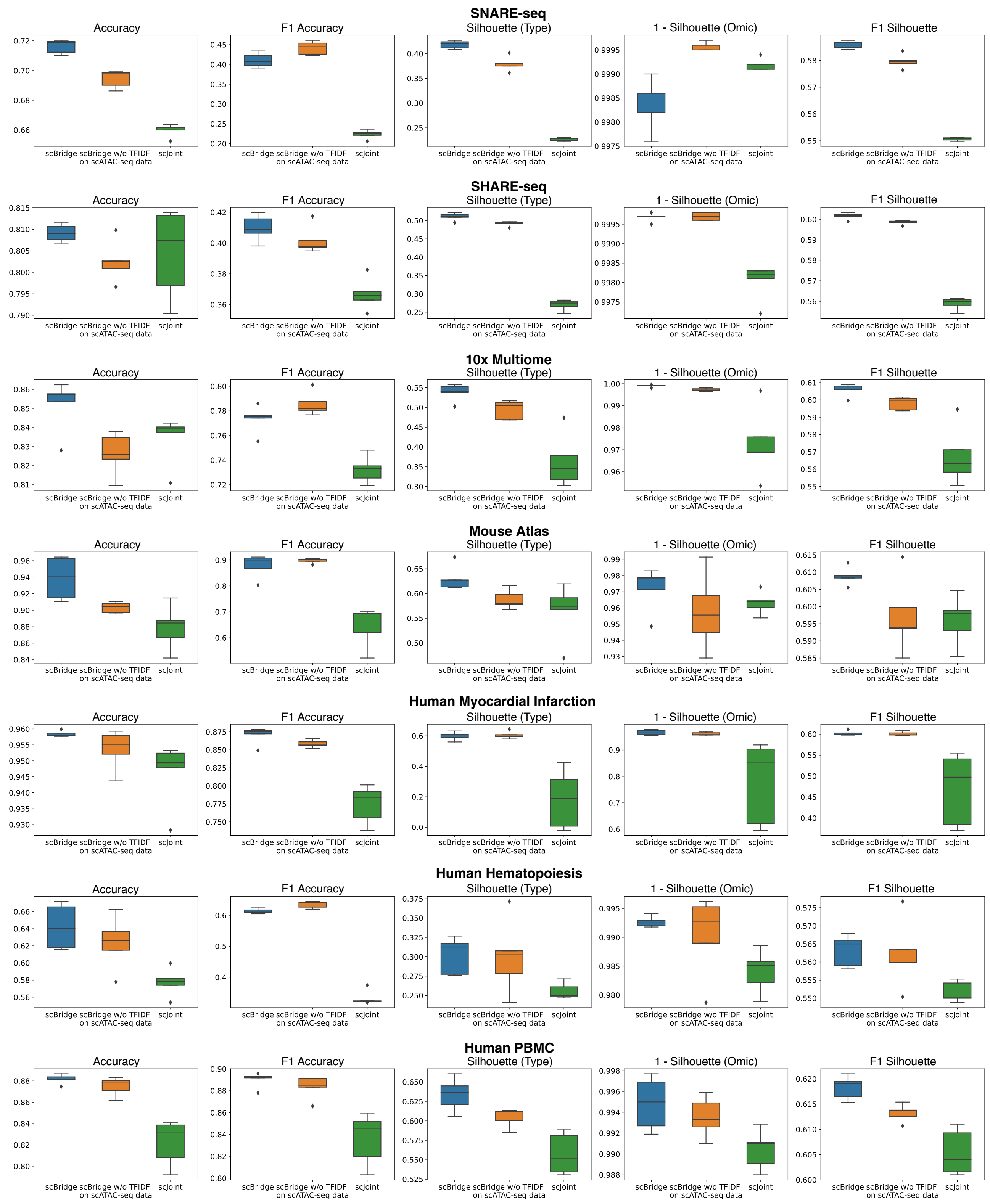

Supplementary Figure 10. Quantitative results of scBridge with different scATAC-seq data preprocessing strategies, compared with the most competitive baseline scJoint. Each boxplot ranges from the upper and lower quartiles with the median as the horizontal line and whiskers extend to 1.5 times the interquartile range. In the figure, "scBridge w/o TFIDF on scATAC-seq data" denotes that the gene activity matrix is preprocessed like scRNA-seq data, namely, multiplied to have 10,000 counts per cell, log normalized, and scaled to have unit variance and zero means. Source data are provided as a Source Data file.

Supplementary Notes

Supplementary Note 1. scBridge adapts to other modalities.

Though scBridge is designed for scRNA-seq and scATAC-seq data, it also adapts to other modalities as long as the raw input matrices from different modalities are aligned in columns (*i.e.*, genes, proteins, etc.). As a showcase, we applied scBridge to the protein data from the human peripheral blood mononuclear dataset[2], which are obtained by CITE-seq and ASAP-seq, respectively. As illustrated in Fig. 9e, scBridge successfully integrates the protein data from two sources. We quantitatively compared the performance of scBridge and scJoint[3] in terms of integration quality and label transfer accuracy. Results in Fig. 9d and f indicate that scBridge achieves better performance, which proves the potential and flexibility of scBridge in handling different modalities.

Supplementary Note 2. How and when to discover novel types?

Here we supplied the detailed pipeline of novel type discovery. After the training with the structure preservation loss L_{STC}, scBridge would compute a confidence score ranged $[0,1]$ for each of the n^t scATAC-seq cells, denoted as $r^t = \{r_1^t, r_2^t, \ldots, r_{n^t}^t\}$. To predict which portion of cells is novel, we derived a confidence threshold in a data-driven manner. Specifically, we fit the distribution of r^t with a two-component GMM g, namely,

$$g\left(r^t\right) = \gamma_{c_1}\,\phi\left(d^t \mid c_1\right) + \gamma_{c_2}\,\phi\left(d^t \mid c_2\right), \tag{1}$$

where $\gamma_{c_1}, \gamma_{c_2}$ denote the mixture coefficient for components c_1, c_2. Let $p(c_1 \mid r_i^t), p(c_2 \mid r_i^t) \in [0,1]$ be the probability of cell i belonging to the two components, the confidence threshold dividing novel and seen cell types is estimated as β that satisfies $p(c_1 \mid r_i^t) = p(c_2 \mid r_i^t)$. If there is more than one suitable $\beta \in [0,1]$, we chose the smallest one as the confidence threshold. Finally, those scATAC-seq cells with $r_i^t < \beta$ are predicted as novel. The confidence score distribution, the two GMM components, and the estimated confidence threshold of scBridge and other baselines are illustrated in Fig. 4b and Supplementary Fig. 6e.

Next, to handle the occasion where users do not have enough prior information to decide whether there are novel cell types in scATAC-seq data, we provided guidance on when to enable the structure preservation loss of scBridge for novel type discovery. Specifically, we encourage the users to run scBridge twice with and without the structure preservation loss respectively, and visualize the scATAC-seq cell embedding colored by the confidence score. As illustrated in Supplementary Fig. 6f, when there are indeed novel scATAC-seq cells, enabling the structure preservation loss would form a clear cluster of unconfident cells, compared with the UMAP visualization when the loss is disabled (see the left two figures). On the contrary, when there are no novel scATAC-seq cells, only slight differences would be observed between the UMAP visualization with and without the structure preservation loss. Namely, unconfident cells are commonly positioned at the boundary of clusters (see the right two figures). In summary, users could decide whether to enable novel type discovery by observing the pattern differences of unconfident cells with and without the structure preservation loss. Notably, the structure preservation loss intrinsically enables scBridge to discover a portion of cells that are most different from annotated scRNA-seq cells, which is practically useful aided with additional manual analysis on those cells.

Supplementary Note 3. Different preprocessing strategies for scATAC-seq data.

We noted that Seurat[4] preprocesses the scATAC-seq peak data with TF-IDF transformation, which both normalizes across cells to correct for differences in cellular sequencing depth, and across peaks to give higher values to more rare peaks. Motivated by its success, we tried the TF-IDF preprocessing for the gene activity matrix and found it produces promising results as well.

To provide a comprehensive investigation on the TF-IDF preprocessing, we evaluated scBridge with TF-IDF and RNA-like preprocessing for scATAC-seq data. According to the five quantitative metrics on seven datasets shown in Fig. 10, scBridge with TF-IDF preprocessing achieves better performance in most cases than with RNA-like preprocessing. In addition, no matter which preprocessing strategy is adopted, scBridge still outperforms the most competitive baseline scJoint.

Supplementary Note 4. The overall algorithm of scBridge.

Algorithm 1: The scBridge method.

Input: scRNA-seq data X^s; scRNA-seq annotation Y^s; scATAC-seq data X^t; deep embedding network $f(\cdot)$; classification head $g(\cdot)$; warm-up epochs E_1; training epochs E_2; batch size N; threshold α.
Output: scATAC-seq data annotation $\hat{Y}^t$.

```
// Training
while X̃^t ≠ ϕ do
    randomly initialize f(·) and g(·)
    // Warm-up
    for epoch = 1 to E_1 do
        sample a mini-batch {x_i^s}_{i=1}^N from X^s
        compute L_WCE in Eq. 1
        update f(·), g(·) to minimize L_WCE through stochastic gradient descent (SGD)
    end
    // Reliability modeling and cross-omics prototype alignment
    for epoch = 1 to E_2 do
        apply Gaussian Mixture Model (GMM) according to Eq. 2–4
        sample a mini-batch {x_i^s}_{i=1}^N from X^s
        compute L_WCE via Eq. 1
        sample a mini-batch {x_i^t}_{i=1}^N from X^t
        update prototypes via Eq. 6
        compute L_ALN via Eq. 7
        update f(·) and g(·) to minimize L_Bridge via Eq. 9 with stochastic gradient descent (SGD)
    end
    // Heterogeneous transfer learning
    select most reliable scATAC-seq cells X̃^t with p(c_2^a | d_i^t) > α and p(c_1^b | l_i^t) > α
    build new annotated dataset X^s = X^s ∪ X̃^t and unlabeled dataset X^t = X^t \ X̃^t
end
// Inference
compute embeddings for both scRNA-seq and scATAC-seq data via H^s = f(X^s) and H^t = f(X^t)
```

References

1. Chen, S., Lake, B. B. & Zhang, K. High-throughput sequencing of the transcriptome and chromatin accessibility in the same cell. *Nat. biotechnology* **37**, 1452–1457, DOI: 10.1038/s41587-019-0290-0 (2019).

2. Mimitou, E. P. *et al.* Scalable, multimodal profiling of chromatin accessibility, gene expression and protein levels in single cells. *Nat. biotechnology* **39**, 1246–1258 (2021).

3. Lin, Y. *et al.* scjoint integrates atlas-scale single-cell rna-seq and atac-seq data with transfer learning. *Nat. Biotechnol.* **40**, 703–710 (2022).

4. Stuart, T. *et al.* Comprehensive integration of single-cell data. *Cell* **177**, 1888–1902 (2019).
