# Supplementary material for: scBridge embraces cell heterogeneity in single-cell RNA-seq and ATAC-seq data integration
Source: Nat Commun. 2023 Sep 28;14:6045. doi: 10.1038/s41467-023-41795-5 (PMC10539354; doi:10.1038/s41467-023-41795-5)
Supplement: Supplementary file 3 — Reporting Summary [file 41467_2023_41795_MOESM3_ESM.pdf]

nature portfolio

Corresponding author(s): Xi Peng

Last updated by author(s): Aug 28, 2023

Reporting Summary

Nature Portfolio wishes to improve the reproducibility of the work that we publish. This form provides structure for consistency and transparency in reporting. For further information on Nature Portfolio policies, see our Editorial Policies and the Editorial Policy Checklist.

Statistics

For all statistical analyses, confirm that the following items are present in the figure legend, table legend, main text, or Methods section.

n/a	Confirmed	
☐	☒	The exact sample size (*n*) for each experimental group/condition, given as a discrete number and unit of measurement
☐	☒	A statement on whether measurements were taken from distinct samples or whether the same sample was measured repeatedly
☐	☒	The statistical test(s) used AND whether they are one- or two-sided<br>*Only common tests should be described solely by name; describe more complex techniques in the Methods section.*
☒	☐	A description of all covariates tested
☐	☒	A description of any assumptions or corrections, such as tests of normality and adjustment for multiple comparisons
☐	☒	A full description of the statistical parameters including central tendency (e.g. means) or other basic estimates (e.g. regression coefficient) AND variation (e.g. standard deviation) or associated estimates of uncertainty (e.g. confidence intervals)
☐	☒	For null hypothesis testing, the test statistic (e.g. *F*, *t*, *r*) with confidence intervals, effect sizes, degrees of freedom and *P* value noted<br>*Give P values as exact values whenever suitable.*
☒	☐	For Bayesian analysis, information on the choice of priors and Markov chain Monte Carlo settings
☒	☐	For hierarchical and complex designs, identification of the appropriate level for tests and full reporting of outcomes
☐	☒	Estimates of effect sizes (e.g. Cohen's *d*, Pearson's *r*), indicating how they were calculated

Our web collection on statistics for biologists contains articles on many of the points above.

Software and code

Policy information about availability of computer code

Data collection	No software was used for data collection.
Data analysis	scBridge is available at https://github.com/XLearning-SCU/scBridge.The following packages were used for processing and data analysis: PyTorch (v1.12.1), Scikit-learn (v.1.1.1), bwa (v0.7.17-r1198-dirty19), Signac (v1.8.0), ArchR (v1.0.2), scJoint (https://github.com/SydneyBioX/scJoint), Seurat (v4.1.4), Portal (v1.0.2), Harmonypy (v0.0.5), GLUE (v0.3.2), and Conos (v1.4.6), monocle (v2.22.0), Scanpy (v1.9.1), ggplot2 (v3.4.1), seaborn (v0.11.2), SciPy (v1.11.1).

For manuscripts utilizing custom algorithms or software that are central to the research but not yet described in published literature, software must be made available to editors and reviewers. We strongly encourage code deposition in a community repository (e.g. GitHub). See the Nature Portfolio guidelines for submitting code & software for further information.

Data

Policy information about availability of data

All manuscripts must include a data availability statement. This statement should provide the following information, where applicable:

- Accession codes, unique identifiers, or web links for publicly available datasets
- A description of any restrictions on data availability
- For clinical datasets or third party data, please ensure that the statement adheres to our policy

All datasets used in this work are publicly available.
(1) Mouse SNARE-seq cortex data used in this study are available in the GEO database with accession ID GSE126074 (https://www.ncbi.nlm.nih.gov/geo/query/acc.cgi?acc=GSE126074)
(2) Human SHARE-seq BMMC data used in this study are available in the GEO database with accession ID GSE207308 (https://www.ncbi.nlm.nih.gov/geo/query/acc.cgi?acc=GSE207308).
(3) Mouse 10x Multiome kidney data used in this study could be downloaded from https://www.10xgenomics.com/resources/datasets/mouse-kidney-nuclei-isolated-with-chromium-nuclei-isolation-kit-saltyez-protocol-and-10x-complex-tissue-dp-ct-sorted-and-ct-unsorted-1-standard.
(4) Mouse atlas scRNA-seq data used in this study are available in Figshare (https://figshare.com/projects/Tabula_Muris_Transcriptomic_characterization_of_20_organs_and_tissues_from_Mus_musculus_at_single_cell_resolution/27733). Mouse atlas scATAC-seq data used in this study are available in the GEO database with accession ID GSE111586 (https://www.ncbi.nlm.nih.gov/geo/query/acc.cgi?acc=GSE111586) and https://atlas.gs.washington.edu/mouse-atac/.
(5) Human myocardial infarction data used in this study are available in zenodo databases with accession ID 6578553 (https://zenodo.org/record/6578553), 6578617 (https://zenodo.org/record/6578617), and https://cellxgene.cziscience.com/collections/8191c283-0816-424b-9b61-c3e1d6258a77.
(6) Human hematopoiesis data could be downloaded from https://github.com/GreenleafLab/MPAL-Single-Cell-2019.
(7) Human PBMC data could be downloaded from https://github.com/SydneyBioX/scJoint/blob/main/data.zip.

Research involving human participants, their data, or biological material

Policy information about studies with human participants or human data. See also policy information about sex, gender (identity/presentation), and sexual orientation and race, ethnicity and racism.

Reporting on sex and gender	All the data used in our study are publicly available. This information has not been collected.
Reporting on race, ethnicity, or other socially relevant groupings	All the data used in our study are publicly available. This information has not been collected.
Population characteristics	All the data used in our study are publicly available. This information has not been collected.
Recruitment	All the data used in our study are publicly available. This information has not been collected.
Ethics oversight	All the data used in our study are publicly available. This information has not been collected.

Note that full information on the approval of the study protocol must also be provided in the manuscript.

Field-specific reporting

Please select the one below that is the best fit for your research. If you are not sure, read the appropriate sections before making your selection.

☒ Life sciences ☐ Behavioural & social sciences ☐ Ecological, evolutionary & environmental sciences

For a reference copy of the document with all sections, see nature.com/documents/nr-reporting-summary-flat.pdf

Life sciences study design

All studies must disclose on these points even when the disclosure is negative.

Sample size	No statistical methods were used to predetermine sample size. We evaluated our method on three golden benchmarks with ground-truth pairing information for data integration evaluation, including Mouse SNARE-seq cortex data, Human SHARE-seq BMMC data, and Mouse 10x Multiome kidney data. We also evaluated our method on four other datasets, including Mouse atlas data, Human myocardial infarction data, Human hematopoiesis data, and Human peripheral blood mononuclear cells, which cover different tissues, species, number of cell types, sequencing technologies, and platforms. We further conducted downsampling and dropout simulations to investigate the robustness of our method against the number of cells and sequencing depths. In other words, the datasets used in our evaluation are sufficient to demonstrate the effectiveness and robustness of our method.
Data exclusions	Standard filtering procedures were applied to exclude low-quality cells and low-count genes. The details can be found in the method section.
Replication	All the computational results were replicable by running our algorithm five times using independent random initializations.

Randomization	Our study does not involve allocating samples to experimental groups.
Blinding	Our study does not involve group allocation that requires blinding.

Reporting for specific materials, systems and methods

We require information from authors about some types of materials, experimental systems and methods used in many studies. Here, indicate whether each material, system or method listed is relevant to your study. If you are not sure if a list item applies to your research, read the appropriate section before selecting a response.

Materials & experimental systems

n/a	Involved in the study
☒	☐ Antibodies
☒	☐ Eukaryotic cell lines
☒	☐ Palaeontology and archaeology
☒	☐ Animals and other organisms
☒	☐ Clinical data
☒	☐ Dual use research of concern
☒	☐ Plants

Methods

n/a	Involved in the study
☒	☐ ChIP-seq
☒	☐ Flow cytometry
☒	☐ MRI-based neuroimaging
